# Selective Anterior Fixation for Rami Fractures in Anteroposterior Compression-Type Pelvic Ring Injuries: Impact of Posterior Stability

**DOI:** 10.3390/jcm15103773

**Published:** 2026-05-14

**Authors:** Jeong-Hyun Koh, Sumin Lim, Won-Tae Cho, Seungyeob Sakong, Wan-Sun Choi, Daehyun Han, Hyung Keun Song

**Affiliations:** Department of Orthopedic Surgery, Ajou University School of Medicine, Suwon 16499, Republic of Korea; osboy513@gmail.com (J.-H.K.); khoo1003@aumc.ac.kr (S.L.); ccarius85@gmail.com (W.-T.C.); sgsy4040@gmail.com (S.S.); cws517@hanmail.net (W.-S.C.); eogus614@gmail.com (D.H.)

**Keywords:** pelvic fractures, pubic symphysis, fracture fixation, internal, pelvic ring

## Abstract

**Background/Objectives**: Rami fractures in anteroposterior compression (APC)-type pelvic ring injuries show favorable outcomes with conservative management in isolated settings; however, the necessity of direct rami fixation when posterior instability is present remains unclear. This study aimed to determine whether adding direct rami fixation to symphyseal plating improves clinical and radiologic outcomes in APC-type pelvic ring injuries. **Methods**: This retrospective cohort study included a final cohort of 98 patients with APC type II or III pelvic ring injuries and concomitant pubic rami fractures treated at a Level 1 trauma center (2014–2022). All patients underwent plate-based symphyseal fixation, classified into four groups by fixation strategy. Primary outcomes were rami nonunion and implant-related complications, analyzed with parsimonious multivariate logistic regression (events-per-variable ratio ≥ 10). **Results**: Among 98 patients (mean age 45.4 ± 16.2 years; 76.5% male), complete posterior ring injury was independently associated with rami nonunion (aOR 8.176; 95% CI 2.448–27.309; *p* = 0.001), implant-related complications (aOR 3.364; 95% CI 1.250–9.049; *p* = 0.016), and overall complications (aOR 4.292; 95% CI 1.640–11.233; *p* = 0.003). Female sex was an additional independent predictor of overall complications (aOR 4.226; 95% CI 1.443–12.378; *p* = 0.009). Direct rami fixation was not a significant predictor of any outcome but consistently increased operative time in pairwise subgroup comparisons (Group 1 vs. 2: 64.9 vs. 106.9 min, *p* < 0.001; Group 3 vs. 4: 95.1 vs. 153.5 min, *p* < 0.001). Pairwise subgroup comparisons were severely underpowered (power range 5–16%); therefore, the absence of statistically significant differences between fixation strategies should not be interpreted as evidence of equivalence. Because more complex fractures were more likely to receive additional fixation, confounding by indication further limits these comparisons. **Conclusions**: Complete posterior ring injury was the dominant predictor of adverse outcomes in APC-type pelvic ring injuries. In this underpowered exploratory analysis, adding direct rami fixation to symphyseal plating did not demonstrate a statistically significant reduction in complications but was associated with longer operative time. Direct rami fixation may be reserved for selected cases with marked displacement, poor indirect reduction, or compromised bone quality; larger prospective studies are needed before firm recommendations can be made.

## 1. Introduction

Pelvic ring injuries, often resulting from high-energy trauma, are associated with substantial morbidity and mortality [1]. Among these, anteroposterior compression (APC)-type injuries typically manifest as pubic symphysis diastasis accompanied by superior and/or inferior pubic rami fractures [2]. Rami fractures exhibit high union rates of up to 96.5% and favorable functional outcomes with conservative management alone in cases of low-energy or isolated injuries [3]. However, in the context of unstable pelvic ring injuries, the clinical significance and optimal surgical management of rami fractures remain uncertain, with no clear consensus on the necessity of fixation.

Previous studies have established surgical indications for APC injuries based on the Young–Burgess classification [4] or the magnitude of symphyseal diastasis [5]. It is widely recognized that posterior ring stabilization is crucial for restoring overall pelvic stability and reducing the failure of anterior fixation [6,7]. Nevertheless, the necessity of direct fixation for concomitant rami fractures, once the posterior component has been adequately addressed, remains inadequately investigated [8]. While some reports suggest high union rates for rami fractures without additional fixation when the posterior ring is stabilized, evidence for generalizing this approach to APC-type injuries remains insufficient [9]. In particular, while biomechanical and clinical studies have examined the role of posterior fixation in APC-type injuries, comparative studies evaluating the incremental benefit of direct rami fixation after both symphyseal plating and posterior ring stabilization remain lacking [10,11,12].

Furthermore, the Young–Burgess classification provides a useful organizational framework, but APC-type injuries, particularly APC-II patterns, may demonstrate substantial within-classification heterogeneity in posterior pelvic ring disruption. Injuries classified as APC-II may range from minimally displaced patterns with intact posterior ligaments to near-complete posterior ligamentous disruption, creating a clinical spectrum within a single classification category that may explain variable outcomes even among patients categorized identically [13,14]. Surgical fixation of rami fractures requires additional soft tissue dissection and implant placement, potentially increasing operative time, blood loss, and the risk of wound complications or infection [15,16]. Conversely, performing symphyseal plating alone may simplify the procedure and reduce morbidity, particularly in older or medically fragile patients. However, concerns persist that omitting direct rami fixation could compromise stability or increase the risk of nonunion and implant-related complications in certain injury patterns, particularly in the presence of substantial posterior ring injury [6].

Therefore, clinical evidence specifically evaluating the necessity for direct rami fixation in APC-type pelvic injuries, considering posterior ring stability, is needed. This study aimed to retrospectively analyze the clinical and radiologic outcomes of APC-type pelvic ring injuries with concomitant rami fractures, according to differences in the extent of anterior fixation and the status of posterior fixation. The primary objective was to explore whether additional rami fixation is associated with lower rates of rami nonunion and implant-related complications once adequate posterior fixation has been achieved. We hypothesized that, in APC-type pelvic injuries with a stabilized posterior ring, symphyseal plating alone would not be associated with substantially higher rates of rami nonunion or implant-related complications compared with more extensive anterior fixation and that complete posterior ring injury would remain a major determinant of adverse outcomes.

## 2. Materials and Methods

### 2.1. Study Design and Patient Selection

This retrospective cohort study was conducted at a single Level 1 trauma center and included 98 patients who underwent surgical treatment for APC-type pelvic ring injuries with pubic rami fractures between January 2014 and December 2022. During this period, all consecutive patients with APC type II or III pelvic ring injuries and at least one pubic ramus fracture were screened for eligibility.

The inclusion criteria were as follows: (1) APC type II or III pelvic ring injury according to the Young–Burgess classification and (2) the presence of at least one pubic rami fracture. Patients were excluded if they had sacral fractures or crescent fractures of the ilium involving the posterior iliac wing. Exclusion criteria were fragility or pathological fractures, periprosthetic fractures, in-hospital death before definitive fixation, less than 24 months of clinical and radiologic follow-up, and incomplete medical or radiologic records.

To reduce the heterogeneity of fixation constructs and minimize bias related to different surgical techniques, we further excluded patients in whom pubic rami fractures were treated with isolated retrograde or antegrade ramus screws, with or without a separate symphyseal plate, or with anterior fixation methods other than plate-based symphyseal fixation. For the posterior ring, we excluded patients treated with spinopelvic fixation, posterior tension band plating, or other constructs beyond iliosacral screw fixation. Only patients whose posterior ring was stabilized with one or two cannulated iliosacral screws at the S1 and/or S2 levels were included in the analysis.

After applying all inclusion and exclusion criteria, 98 patients with APC type II or III pelvic ring injuries and concomitant pubic rami fractures treated with plate-based symphyseal fixation (with or without iliosacral screw fixation) were included in the final analysis. All consecutive patients meeting the aforementioned criteria during the study period were included.

### 2.2. Data Collection and Radiologic Evaluation

We collected demographic, injury-related, clinical, and radiological data from the hospital’s electronic medical record database and the picture archiving and communication system (INFINITT PACS, version 3.0.11.3; INFINITT Healthcare, Seoul, Republic of Korea). Radiographic variables included the Young–Burgess classification, Nakatani classification [11] of rami fractures, initial rami fracture gap (mm), rami nonunion, implant-related complications, and overall complication rates. Rami nonunion was defined according to the criteria used by Yoon et al. [3], as the absence of complete bone union at the fracture site on anteroposterior, inlet, and outlet radiographs and/or computed tomography (CT) obtained more than 6 months after surgery, or a lack of radiographic progression of union on serial radiographs over a period of at least 3 months, combined with persistent local pain or functional limitation at the fracture site during weight-bearing.

Complete posterior ring injury is characterized by disruption of the posterior pelvic arch, resulting in both rotational and vertical instability. This corresponds to ligamentous Tile type C patterns involving complete sacroiliac joint disruption without associated sacral or iliac wing fractures (sacral and crescent fractures were excluded by the study criteria). Radiologic reduction quality of pubic rami fractures was assessed using the Tornetta criteria [17] and graded as Excellent (anatomic reduction), Good (<2 mm residual displacement), Fair (2–5 mm displacement), or Poor (>5 mm displacement); grades of Good or Excellent were considered an acceptable reduction. All radiographs and CT scans were independently evaluated by board-certified orthopedic trauma surgeons [18]. For the final cohort, all variables necessary for the primary and secondary analyses were available from the medical records and PACS, thus obviating the need for imputation of missing data.

### 2.3. Surgical Strategy

Posterior pelvic ring fixation was performed in patients with mechanical instability, as confirmed by preoperative CT imaging and intraoperative examination under anesthesia [2,19]. In this cohort, to maintain homogeneity of fixation constructs, posterior stabilization was achieved exclusively with one or two 7.3 mm cannulated iliosacral screws (DePuy Synthes, Zuchwil, Switzerland) inserted percutaneously at the S1 and/or S2 levels. Screw trajectory and level (S1 and/or S2) were selected based on fracture morphology and bone stock, following conventional techniques under fluoroscopic guidance.

Anterior ring fixation was not randomized but was performed selectively according to the attending surgeon’s judgment and fracture characteristics. During the study period, three to four board-certified orthopedic trauma surgeons (each with more than 5 years of independent practice in pelvic trauma surgery) performed the procedures, each applying individual clinical judgment without a standardized institutional protocol for the decision to add direct rami fixation. In general, direct rami fixation was considered when significant rami displacement, comminution, bilateral fracture pattern, or insufficient indirect reduction remained after symphyseal plating and posterior stabilization; however, these criteria were not formally defined or consistently applied across surgeons. Consequently, the allocation to each fixation strategy was observational rather than randomized and is inherently susceptible to selection bias and confounding by indication. Specifically, surgeons were more likely to select direct rami fixation for more displaced, comminuted, or otherwise complex fracture patterns, which may bias comparisons between fixation strategies. All patients underwent plate-based symphyseal fixation.

For anterior fixation, all symphyseal plating procedures were performed through a transverse Pfannenstiel incision. When only the pubic symphysis required fixation, a precontoured pelvic plate (Low Profile 3.5 mm Pelvic System^®^, DePuy Synthes, Zuchwil, Switzerland) was applied to the anterior surface of the symphysis according to standard technique. When direct fixation of the pubic rami was planned in addition to symphyseal fixation, the same Pfannenstiel incision was used to perform a modified Stoppa approach, providing extraperitoneal exposure of the pubic symphysis and superior pubic ramus. In these cases, a single long 3.5-mm reconstruction plate (DePuy Synthes, Zuchwil, Switzerland) was contoured to span the pubic symphysis and the affected pubic ramus (or rami), allowing simultaneous stabilization of both the symphysis and the rami through one construct.

Based on the final surgical fixation strategy, patients were classified into four groups: Group 1, symphyseal plating alone without posterior fixation; Group 2, symphyseal plating with direct rami fixation without posterior fixation; Group 3, symphyseal plating with iliosacral screw fixation of the posterior ring; and Group 4, symphyseal plating with direct rami fixation and iliosacral screw fixation of the posterior ring.

### 2.4. Statistical Analysis

All statistical analyses were conducted using the SPSS software (version 29.0; IBM Corp., Armonk, NY, USA). Continuous variables were compared across the four groups using the Kruskal–Wallis test, while categorical variables were assessed using the chi-square or Fisher’s exact test, as appropriate. Planned pairwise comparisons (Group 1 vs. 2 and Group 3 vs. 4) were performed using the Mann–Whitney U test or Fisher’s exact test to evaluate the effect of ramus fixation relative to the posterior fixation status.

Logistic regression was employed to identify predictors of rami nonunion, implant-related complications, and overall complications. Initially, univariate analyses were performed to screen potential predictors, and variables with *p* < 0.15 were considered candidates for multivariate models. Clinically relevant variables examined in univariate analysis included age, sex, BMI, APC type (II vs. III), complete posterior ring injury, initial rami fracture gap, bilateral rami fractures, ISS score, Tornetta reduction grade (acceptable vs. not), and the use of posterior fixation and direct rami fixation. Given the limited number of outcome events (19 nonunions, 22 implant-related complications, and 31 overall complications), parsimonious multivariate models were constructed to maintain an events-per-variable (EPV) ratio of at least 10, which is the conventionally recommended minimum to avoid model overfitting. Accordingly, two mandatory covariates were included in all models: complete posterior ring injury (the primary clinical predictor of interest) and direct rami fixation (the primary intervention variable). A third covariate, female sex, was additionally included in the overall complication model, as it reached statistical significance in univariate analysis and the EPV ratio permitted inclusion (EPV = 10.3). APC type III, although significant in univariate analysis for some outcomes, was not included in multivariate models because of its collinearity with complete posterior ring injury and EPV constraints. A post-hoc power analysis was performed using the observed event rates and effect sizes to estimate the achieved statistical power of the primary comparisons. Results were reported as adjusted odds ratios (aORs) with 95% confidence intervals (CIs). A two-sided *p* < 0.05 was considered statistically significant. Given the retrospective, non-randomized design and the exploratory nature of the multivariate models, all results should be interpreted as hypothesis-generating rather than confirmatory. Based on the observed event rates, achieving 80% statistical power to detect a clinically meaningful difference of 10–15 percentage points in complication rates would require substantially larger sample sizes (approximately 180–220 patients per treatment group; α = 0.05, two-tailed), far exceeding the current sample.

### 2.5. Ethics Approval

This study was approved by the Institutional Review Board of Ajou University (Approval Number: AJOUIRBDB2025371). The study was conducted in accordance with the Declaration of Helsinki and local regulatory requirements. The requirement for informed consent was waived owing to the retrospective nature of the study.

## 3. Results

After applying all inclusion and exclusion criteria, a final cohort of 98 patients was included in the analysis. The mean rami fracture gap was 5.95 mm (standard deviation [SD] ± 5.39), and the average operation time was 106.64 min (SD ± 49.48). According to the Young–Burgess classification, 72.4% of the patients had type II APC and 27.6% had type III. Radiologic reduction quality was acceptable (Good or Excellent) in 87 patients (88.8%); the remaining 11 (11.2%) were graded as Fair or Poor. The rates of rami nonunion, implant-related complications (loosening, breakage, or infection), and overall complications were 19.4%, 22.4%, and 31.6%, respectively (Table 1). Among the 71 APC-II patients, 13 (18.3%) had complete posterior ring injury despite being classified as APC-II, whereas the remaining 58 (81.7%) had incomplete posterior ring injury. All 27 APC-III patients (100%) had complete posterior ring injury. This distribution indicates that a notable minority of APC-II injuries demonstrate posterior ring disruption beyond what the classification category implies, supporting the concept of within-classification heterogeneity.

Complete posterior ring injury was independently associated with adverse outcomes across all three parsimonious multivariate models. It significantly increased the odds of rami fracture nonunion (aOR 8.176, 95% CI 2.448–27.309, *p* = 0.001; Table 2), implant-related complications (aOR 3.364, 95% CI 1.250–9.049, *p* = 0.016; Table 3), and overall complications (aOR 4.292, 95% CI 1.640–11.233, *p* = 0.003; Table 4). Female sex was additionally identified as an independent predictor of overall complications (aOR 4.226, 95% CI 1.443–12.378, *p* = 0.009; Table 4). Baseline characteristics did not differ significantly between male (*n* = 75) and female (*n* = 23) patients with respect to age (45.8 ± 14.4 vs. 44.3 ± 21.3 years; *p* = 0.348), BMI (26.0 ± 4.0 vs. 24.8 ± 4.7 kg/m^2^; *p* = 0.500), ISS (33.5 ± 11.4 vs. 33.3 ± 13.1; *p* = 0.903), rami fracture gap (5.7 ± 5.4 vs. 6.7 ± 5.2 mm; *p* = 0.426), APC type III proportion (28.0% vs. 26.1%; *p* = 1.000), or complete posterior ring injury rate (42.7% vs. 34.8%; *p* = 0.629). The overall complication rate was significantly higher in female patients (52.2% vs. 25.3%; *p* = 0.021), consistent with the multivariate finding. Implant-related complications (34.8% vs. 18.7%; *p* = 0.151) and rami nonunion (26.1% vs. 17.3%; *p* = 0.374) showed numerically higher rates in female patients but did not reach statistical significance individually. Direct rami fixation was not a statistically significant predictor of any outcome in multivariate analysis. No other demographic, injury-related, or radiologic variables showed statistically significant associations in the multivariate models.

There were no statistically significant differences in demographic characteristics among the subgroups (Table 5). Tornetta reduction quality did not differ significantly across the four groups overall (*p* = 0.139); however, the proportion of acceptable reductions was lower in Group 4 (74.1%) compared with Group 3 (96.3%), with a borderline significant difference (Fisher’s exact test, *p* = 0.050; Table 5). Reduction quality (Tornetta acceptable) was not an independent predictor of any primary outcome in univariate logistic regression analysis (nonunion: OR 0.365, *p* = 0.143; implant-related complications: OR 0.745, *p* = 0.685; overall complications: OR 0.788, *p* = 0.721; Table 2, Table 3 and Table 4), and was therefore not included in the multivariate models. Given the severely limited statistical power of pairwise subgroup comparisons (5–16%), the following results should be interpreted with caution. Subgroup analysis revealed that direct rami fixation resulted in a significantly longer operation time in patients without (Group 1 vs. Group 2, *p* < 0.001) and with (Group 3 vs. Group 4, *p* < 0.001) posterior fixation (Table 6). However, no significant differences were observed in rami nonunion, implant-related complications, or overall complication rates between the groups (Table 6). Post-hoc power analysis revealed that these pairwise comparisons were severely underpowered (power range 5–16%), precluding definitive conclusions regarding the equivalence of fixation strategies for these outcomes. Other clinical variables, such as intensive care unit stay, ventilator days, admission duration, and time to surgery, did not differ significantly across the subgroups (Table 5 and Table 6).

## 4. Discussion

This study evaluated the impact of direct pubic rami fixation in APC-type pelvic ring injuries combining symphyseal rupture, pubic rami fractures, and posterior ring disruption. The principal finding was that, in parsimonious multivariate models, complete posterior ring injury was consistently and independently associated with rami nonunion, implant-related complications, and overall complications after adjustment for other covariates. Female sex was additionally identified as an independent predictor of overall complications. In contrast, adding direct rami fixation to symphyseal plating was not associated with a statistically significant reduction in rami nonunion or implant-related complications in either the anterior-only or posterior-fixation strata, but it was consistently associated with significantly longer operative times. Therefore, in this exploratory cohort analysis, routinely adding direct rami fixation after symphyseal plating and posterior stabilization was not associated with a statistically significant reduction in complications. However, the severely limited statistical power precludes any inference of equivalence between fixation strategies.

Although APC type II injuries are classically described as having incomplete posterior ring disruption with intact posterior sacroiliac ligaments [4,20], previous biomechanical and clinical studies have indicated that they can still exhibit substantial residual posterior instability and high rates of anterior plate failure when managed with anterior fixation alone [6]. These data suggest that even “incomplete” posterior injuries in APC type II patterns may behave more like a spectrum of posterior ligamentous disruption than a truly stable posterior arch. The Young–Burgess APC type II classification encompasses a clinical spectrum rather than a discrete anatomic entity: at one end, minimally displaced injuries with preserved posterior ligamentous tension may behave as functionally stable patterns, while at the other end, injuries with near-complete posterior ligamentous disruption approach the instability profile of APC type III. In our cohort, 18.3% of APC-II patients were found to have complete posterior ring injury, quantitatively demonstrating this within-classification heterogeneity and supporting the need for individualized assessment of posterior ring integrity beyond classification alone [13]. Indeed, nondisplaced or subtle fractures accompanying the primary injury pattern can be easily overlooked in the pelvis and lower extremity [21], further underscoring the importance of thorough evaluation of the posterior ring in APC-type injuries. However, these observations are descriptive and should be interpreted cautiously given the limited sample size. This within-classification heterogeneity means that not all APC type II injuries carry equivalent risk, and that blanket treatment approaches based solely on Young–Burgess type may be insufficient; additional assessment of posterior ligamentous integrity—through stress radiography, dynamic fluoroscopy, or intraoperative examination under anesthesia—may therefore be necessary to stratify patients more accurately [6]. Our findings align with this concept: all patients treated with anterior fixation alone in this study (Groups 1 and 2) had APC type II injuries, yet they still experienced non-negligible rates of rami nonunion and implant-related complications. This observation is consistent with previous reports indicating that APC type II injuries are prone to failure when the posterior ring is not adequately addressed and supports the growing view that posterior ring fixation should be strongly considered even in selected APC type II injuries, although our study was not specifically designed or powered to define definitive indications for posterior fixation in this subgroup.

The central role of the posterior ring in overall pelvic stability has been emphasized in both biomechanical and clinical literature [10,12]. Posterior fixation of APC-type injuries has been demonstrated to reduce anterior plate failure, decrease malunion rates, and restore rotational and sagittal stability more effectively than anterior fixation alone [22]. Experimental models have demonstrated that, in rotationally unstable open-book injuries, symphyseal plating alone substantially reduces motion at the symphysis but leaves considerable residual motion at the sacroiliac joint, whereas posterior fixation more effectively controls pelvic deformation under physiological loading [23]. Clinical series similarly report that combined anterior–posterior fixation yields lower rates of loss of reduction and implant failure compared to anterior fixation alone [8,9]. In our parsimonious multivariate models, complete posterior ring injury remained significantly associated with all three adverse outcomes, and female sex emerged as an additional independent predictor of overall complications; this further underscores the dominant influence of the posterior ring on the healing of pubic rami fractures and performance of anterior implants. The finding that female sex was an independent predictor of overall complications (aOR 4.226, *p* = 0.009) warrants further investigation. In our sex-stratified descriptive analysis, baseline demographics, injury severity, fracture characteristics, and treatment strategies did not differ significantly between male and female patients, suggesting that the elevated complication risk in female patients was not attributable to measurable differences in injury complexity or treatment approach. Potential explanations include sex-related differences in pelvic anatomy affecting implant fixation, lower bone mineral density particularly in perimenopausal or postmenopausal women, and hormonal influences on fracture healing biology [24,25]. However, menopausal status and bone mineral density were not assessed in this study, and the relatively small number of female patients (*n* = 23) limits the statistical robustness of this finding. Future studies with larger female cohorts and prospective collection of bone quality parameters are needed to clarify the mechanisms underlying sex-specific complication risk in pelvic ring injuries. Further sex-stratified treatment effect analyses or interaction analyses were not performed owing to the limited number of female patients.

From a biomechanical perspective, once posterior ring instability has been treated and deemed stable and the symphysis has been rigidly plated, residual motion and load transfer across the pubic rami may be limited, particularly in injuries with relatively small fracture gaps and simple fracture patterns [23,26]. In our cohort, the mean rami fracture gap was modest and transverse fracture patterns predominated, which is consistent with a relatively stable anterior configuration once the posterior ring and symphysis were stabilized. In our cohort, the absence of a statistically significant reduction in nonunion or implant-related complications with direct rami fixation in our cohort is plausible, because once posterior stability has been restored and the symphysis has been rigidly plated, the incremental mechanical contribution of direct rami fixation may be limited in selected fracture patterns [27]. Nevertheless, given the severely limited statistical power, this finding should not be interpreted as evidence that direct rami fixation has no effect.

These results support a posterior fixation-based treatment strategy for APC-type pelvic ring injuries with combined symphyseal rupture, pubic rami fractures, and posterior ring disruption [5]. Clinically, we advocate addressing posterior instability first, with iliosacral screw fixation when indicated, then stabilizing the symphysis with plate fixation as the default anterior construct [2]. Our data suggest that, after posterior instability had been addressed and deemed stable on CT and examination under anesthesia, symphyseal plating alone was not associated with statistically significant differences in rami nonunion or implant-related complications compared with more extensive anterior constructs, while it was associated with shorter operative time. However, the study was substantially underpowered to detect modest but clinically meaningful treatment effects, and these findings should be interpreted as hypothesis-generating rather than definitive. Accordingly, selective rather than routine use of direct rami fixation appears to be a reasonable strategy for further study. Factors that may favor direct rami fixation include: (1) marked rami displacement (>10 mm) or significant comminution precluding satisfactory indirect reduction through symphyseal plating alone; (2) bilateral rami fractures with substantial fragment gaps on both sides; (3) persistently inadequate reduction after posterior fixation and symphyseal plating (Tornetta Fair or Poor); (4) poor bone stock or osteoporosis compromising implant purchase in the symphyseal plate alone; and (5) young, high-demand patients in whom maximal anterior stability is desirable for early full weight-bearing. Conversely, in patients with small fracture gaps, acceptable indirect reduction (Tornetta Good or Excellent), and adequate bone quality following posterior stabilization, symphyseal plating alone appears to be a reasonable strategy that avoids the additional operative time and soft-tissue dissection associated with direct rami fixation [15,28].

Notably, the non-negligible complication rates observed in APC type II patients treated with anterior fixation alone in our study resonate with prior reports of anterior plate failure and malunion in APC type II injuries managed without posterior fixation [6]. Although our primary aim was not to define the indications for posterior fixation in APC type II injuries, these observations reinforce the notion that posterior instability in APC type II patterns should not be underestimated and that posterior ring fixation should be strongly considered, particularly in the presence of radiologic or intraoperative evidence of posterior laxity [19].

This study possesses several strengths. First, by including only patients with APC type II or III injuries and concomitant pubic rami fractures treated with standardized plate-based symphyseal fixation and, when indicated, iliosacral screw fixation, we focused on a relatively homogeneous subgroup in which fixation constructs were broadly comparable, thereby improving the internal consistency of our comparisons, although this restriction inevitably limits external validity and generalizability to other pelvic injury patterns or fixation methods. Second, by comparing four clinically relevant surgical strategies—symphyseal plating alone, symphyseal plating with direct rami fixation, symphyseal plating with posterior fixation, and combined anterior–posterior fixation with direct rami fixation—we were able to examine the incremental value of rami fixation under conditions that mirror real-world decision-making. Third, the relatively large single-center cohort with a minimum of 24 months of follow-up allowed exploratory multivariate analysis revealing complete posterior ring injury as a variable significantly associated with rami nonunion and complications beyond simple univariate associations.

Some limitations of our study must also be acknowledged. The retrospective single-center design is susceptible to selection bias, and treatment decisions were made at the discretion of the surgeon, rather than based on randomized allocation. Although we attempted to minimize heterogeneity by excluding sacral and crescent fractures and by limiting posterior fixation to iliosacral screws, unmeasured confounders may still have influenced both the choice of fixation strategy and the outcomes. Specifically, surgeons may have been more likely to select direct rami fixation for more displaced, comminuted, or otherwise complex fracture patterns, which could bias comparisons between fixation strategies and potentially obscure any benefit of more extensive anterior constructs. Supporting this concern, the proportion of acceptable radiologic reductions (Tornetta Good or Excellent) was borderline significantly lower in Group 4 than in Group 3 (74.1% vs. 96.3%; *p* = 0.050), suggesting that rami fixation was more frequently performed in cases with more challenging intraoperative reduction, consistent with confounding by indication. This means that observed differences in outcomes between groups may partially reflect differences in baseline fracture complexity rather than treatment efficacy, and any potential benefit of direct rami fixation may have been obscured by its preferential use in more difficult cases. Posterior ring stability was assessed using preoperative CT and intraoperative examination under anesthesia, reflecting current clinical practice but not providing a quantitative biomechanical measure; some residual posterior instability may therefore have gone unrecognized. Future investigations may benefit from incorporating advanced imaging modalities such as stress radiography, dynamic fluoroscopy, or three-dimensional reconstruction technologies [29] to provide more objective quantification of posterior ring stability and fracture complexity. Our primary outcomes comprised radiologic union and implant-related complications. Functional outcomes, pain scores, and patient-reported measures were not systematically collected; hence, subtle differences in function between fixation strategies cannot be excluded. Future prospective studies should incorporate validated patient-reported outcome measures at standardized time points to provide clinically relevant endpoints beyond radiographic and implant-related complications. Finally, the sample size, particularly within subgroups, may have limited the power to detect smaller differences in complication rates, and our findings should be interpreted as hypothesis-generating rather than definitive. Post-hoc power analysis demonstrated that the pairwise subgroup comparisons were severely underpowered (power range 5–16%), meaning that even clinically meaningful differences of 10–15 percentage points in complication rates could not have been detected reliably with the available sample sizes. Consequently, the absence of statistically significant differences between fixation groups should not be interpreted as evidence of equivalence. No formal adjustment for multiple comparisons was performed; therefore, *p*-values for secondary and subgroup analyses should be interpreted with caution.

Despite these limitations, our findings suggest that in APC-type pelvic ring injuries with combined symphyseal rupture, pubic rami fractures, and posterior ring disruption, a posterior fixation-based strategy wherein iliosacral screw fixation and symphyseal plating are routinely performed, whereas direct rami fixation is applied on a case-by-case basis rather than uniformly, may be a reasonable approach within the limitations of this single-center retrospective cohort [30]. Complete posterior ring injury remains a major determinant of rami healing and implant-related complications, highlighting the importance of adequately addressing posterior instability [31]. It should be noted that the absence of observed benefit of direct rami fixation may be biased toward the null due to the limited sample size, residual confounding from non-randomized treatment allocation, and confounding by indication. Future prospective, multicenter studies incorporating detailed functional and patient-reported outcomes and more precise assessments of posterior ring stability are required to confirm these findings and to better identify which patients can safely forgo direct rami fixation after posterior and symphyseal stabilization.

## 5. Conclusions

In this retrospective exploratory analysis of APC-type pelvic ring injuries with combined symphyseal rupture, pubic rami fractures, and posterior ring disruption, complete posterior ring injury was the dominant predictor of rami nonunion, implant-related complications, and overall complications. Adding direct rami fixation to symphyseal plating was not associated with a statistically significant reduction in any outcome measure, but it was associated with significantly longer operative time. However, the severely limited statistical power of pairwise subgroup comparisons substantially constrains the strength of these findings, and the absence of significant differences should not be interpreted as evidence of equivalence between fixation strategies.

These findings support further evaluation of a posterior fixation-based strategy in which posterior instability is addressed with iliosacral screw fixation and the symphysis is plated as the default anterior construct, with selective rather than routine use of direct rami fixation. Larger, adequately powered prospective studies are required before firm treatment recommendations can be made.

## Figures and Tables

**Table 1:** Baseline Demographic, Injury-related, and Perioperative Characteristics of the Study Population (*n* = 98).

Variable	Total (*n* = 98)
Demographics	
Age, years	45.44 ± 16.19
Sex, male/female (n, %)	75/23 (76.5/23.5)
Height, cm	168.89 ± 6.97
Weight, kg	73.61 ± 14.28
BMI, kg/m^2^	25.70 ± 4.21
Smoking, n (%)	54 (55.1)
Injury characteristics	
Injury mechanism, n (%)	
In car-TA	22 (22.4)
Out car-TA	27 (27.6)
Fall	30 (30.6)
Blunt trauma	19 (19.4)
ISS score	33.47 ± 11.76
Initial SBP, mmHg	117.63 ± 24.44
Initial HR, bpm	98.14 ± 24.47
FAST positive, n/N (%)	23/98 (23.5)
Blood transfusion within 24 h, U	6.73 ± 7.22
Fracture characteristics	
Young-Burgess classification, n (%)	
APC type II	71 (72.4)
APC type III	27 (27.6)
Complete posterior ring injury, n (%)	40 (40.8)
Rami fracture side, n (%)	
Unilateral—Right	42 (42.9)
Unilateral—Left	29 (29.6)
Bilateral	27 (27.6)
Nakatani classification, n (%)	
Type 1	2 (2.0)
Type 2	16 (16.3)
Type 3	80 (81.6)
Main rami fracture gap, mm	5.95 ± 5.39
Fixation strategy	
Posterior fixation (iliosacral screw), n (%)	54 (55.1)
Direct rami fixation, n (%)	48 (49.0)
Fixation group, n (%)	
Group 1—Symphyseal plating alone	23 (23.5)
Group 2—Symphyseal plating + rami fixation	21 (21.4)
Group 3—Symphyseal plating + posterior fixation	27 (27.6)
Group 4—Symphyseal + rami + posterior fixation	27 (27.6)
Perioperative data	
Time to surgery, days	3.96 ± 3.29
Operation time, min	106.64 ± 49.48
ICU care duration, days	11.81 ± 12.29
Ventilator care duration, days	5.08 ± 7.47
Length of admission, days	45.77 ± 38.11
Radiologic reduction quality	
Tornetta classification, n (%)	
Excellent	26 (26.5)
Good	61 (62.2)
Fair	10 (10.2)
Poor	1 (1.0)
Acceptable (Good or Excellent), n (%)	87 (88.8)
Primary outcomes	
Rami nonunion, n (%)	19 (19.4)
Implant-related complications, n (%)	22 (22.4)
Overall complications, n (%)	31 (31.6)

Values are presented as mean ± standard deviation or n (%), as appropriate. APC, anteroposterior compression; BMI, body mass index; FAST, focused assessment with sonography for trauma; HR, heart rate; ICU, intensive care unit; ISS, Injury Severity Score; SBP, systolic blood pressure. Tornetta classification: Excellent, anatomic reduction; Good, <2 mm displacement; Fair, 2–5 mm displacement; Poor, >5 mm displacement. Acceptable reduction defined as Good or Excellent. Group 1: symphyseal plating alone (no posterior fixation); Group 2: symphyseal plating + direct rami fixation (no posterior fixation); Group 3: symphyseal plating + iliosacral screw fixation; Group 4: symphyseal plating + direct rami fixation + iliosacral screw fixation.

**Table 2:** Univariate and Multivariate Logistic Regression Analyses of Risk Factors for Pubic Rami Nonunion (Events = 19/98).

Variable	Univariate Analysis		Multivariate Analysis	
	OR (95% CI)	*p*-Value	Adjusted OR (95% CI)	*p*-Value
Age, years	1.014 (0.983–1.046)	0.379		
Female sex	1.683 (0.557–5.088)	0.356		
BMI, kg/m^2^	1.020 (0.907–1.147)	0.741		
APC type III †	3.050 (1.075–8.653)	0.036		
Complete posterior ring injury	8.100 (2.438–26.908)	0.001	8.176 (2.448–27.309)	0.001
Main rami fracture gap, mm	1.064 (0.976–1.160)	0.159		
Bilateral rami fracture	0.925 (0.298–2.874)	0.893		
Posterior fixation	2.008 (0.693–5.815)	0.199		
Direct rami fixation	1.561 (0.567–4.295)	0.389	1.620 (0.542–4.842)	0.388
ISS score	1.002 (0.960–1.046)	0.912		
Reduction quality ‡	0.365 (0.095–1.404)	0.143		

Values are presented as odds ratios (OR) or adjusted odds ratios (aOR) with 95% confidence intervals (CI). Bold values indicate statistical significance (*p* < 0.05). The multivariate model was constructed using a parsimonious approach; complete posterior ring injury and direct rami fixation were included as mandatory covariates (events-per-variable ratio = 9.5).—indicates variable not included in multivariate model. † APC type III was significant on univariate analysis but excluded from multivariate model due to collinearity with complete posterior ring injury. ‡ Reduction quality was assessed using the Tornetta criteria (acceptable = Good or Excellent); it was not a significant predictor in univariate analysis (*p* = 0.143) and was therefore not included in the multivariate model. BMI, body mass index; ISS, Injury Severity Score.

**Table 3:** Univariate and Multivariate Logistic Regression Analyses of Risk Factors for Implant-Related Complications (Events = 22/98).

Variable	Univariate Analysis		Multivariate Analysis	
	OR (95% CI)	*p*-Value	Adjusted OR (95% CI)	*p*-Value
Age, years	0.979 (0.949–1.010)	0.187		
Female sex	2.324 (0.824–6.550)	0.111		
BMI, kg/m^2^	1.008 (0.900–1.127)	0.896		
APC type III †	3.750 (1.378–10.207)	0.010		
Complete posterior ring injury	3.365 (1.251–9.053)	0.016	3.364 (1.250–9.049)	0.016
Main rami fracture gap, mm	0.969 (0.881–1.067)	0.523		
Bilateral rami fracture	2.231 (0.820–6.070)	0.116		
Posterior fixation	1.233 (0.471–3.227)	0.670		
Direct rami fixation	1.054 (0.408–2.723)	0.913	1.035 (0.388–2.759)	0.945
ISS score	1.035 (0.992–1.081)	0.113		
Reduction quality ‡	0.745 (0.180–3.086)	0.685		

Values are presented as odds ratios (OR) or adjusted odds ratios (aOR) with 95% confidence intervals (CI). Bold values indicate statistical significance (*p* < 0.05). Parsimonious multivariate model: events-per-variable ratio = 11.0. † APC type III excluded due to collinearity with complete posterior ring injury. ‡ Reduction quality was assessed using the Tornetta criteria (acceptable = Good or Excellent); it was not a significant predictor in univariate analysis (*p* = 0.685) and was therefore not included in the multivariate model. BMI, body mass index; ISS, Injury Severity Score.

**Table 4:** Univariate and Multivariate Logistic Regression Analyses of Risk Factors for Overall Complications (Events = 31/98).

Variable	Univariate Analysis		Multivariate Analysis	
	OR (95% CI)	*p*-Value	Adjusted OR (95% CI)	*p*-Value
Age, years	0.999 (0.973–1.026)	0.940		
Female sex	3.215 (1.219–8.479)	0.018	4.226 (1.443–12.378)	0.009
BMI, kg/m^2^	0.982 (0.886–1.089)	0.737		
APC type III †	2.189 (0.870–5.512)	0.096		
Complete posterior ring injury	3.468 (1.427–8.430)	0.006	4.292 (1.640–11.233)	0.003
Main rami fracture gap, mm	1.012 (0.935–1.094)	0.773		
Bilateral rami fracture	2.189 (0.870–5.512)	0.096		
Posterior fixation	0.985 (0.418–2.317)	0.972		
Direct rami fixation	1.167 (0.498–2.735)	0.723	0.979 (0.385–2.489)	0.964
ISS score	1.011 (0.975–1.049)	0.547		
Reduction quality ‡	0.788 (0.213–2.918)	0.721		

Values are presented as odds ratios (OR) or adjusted odds ratios (aOR) with 95% confidence intervals (CI). Bold values indicate statistical significance (*p* < 0.05). Parsimonious multivariate model included complete posterior ring injury, direct rami fixation, and female sex (events-per-variable ratio = 10.3). † APC type III excluded due to collinearity with complete posterior ring injury. ‡ Reduction quality was assessed using the Tornetta criteria (acceptable = Good or Excellent); it was not a significant predictor in univariate analysis (*p* = 0.721) and was therefore not included in the multivariate model. BMI, body mass index; ISS, Injury Severity Score.

**Table 5:** Comparison of Baseline Demographic and Injury-related Characteristics among the Four Fixation Strategy Groups.

Variable	Group 1 (*n* = 23)	Group 2 (*n* = 21)	Group 3 (*n* = 27)	Group 4 (*n* = 27)	*p*-Value
Continuous variables—Mean ± SD					
Age, years	44.87 ± 14.77	46.19 ± 18.47	44.00 ± 13.80	46.78 ± 18.31	0.972
BMI, kg/m^2^	25.77 ± 3.23	25.13 ± 3.91	26.13 ± 4.11	25.68 ± 5.31	0.751
ISS score	35.09 ± 11.00	27.38 ± 10.23	35.89 ± 12.56	34.41 ± 11.64	0.061
Blood transfusion, U	3.91 ± 5.25	6.71 ± 6.99	8.15 ± 7.31	7.74 ± 8.37	0.090
Main fracture gap, mm	6.19 ± 7.25	5.50 ± 4.91	5.58 ± 4.92	6.48 ± 4.51	0.785
Categorical variables—n/N (%)					
Male sex	18/23 (78.3%)	13/21 (61.9%)	23/27 (85.2%)	21/27 (77.8%)	0.297
Smoking	15/23 (65.2%)	12/21 (57.1%)	12/27 (44.4%)	15/27 (55.6%)	0.526
FAST positive	5/23 (21.7%)	7/21 (33.3%)	5/27 (18.5%)	6/27 (22.2%)	0.667
Young-Burgess classification, n (%)					
APC type II	23/23 (100%)	21/21 (100%)	14/27 (51.9%)	13/27 (48.1%)	<0.001
APC type III	0/23 (0.0%)	0/21 (0.0%)	13/27 (48.1%)	14/27 (51.9%)	<0.001
Complete posterior ring injury	3/23 (13.0%)	3/21 (14.3%)	17/27 (63.0%)	17/27 (63.0%)	<0.001
Bilateral rami fracture	4/23 (17.4%)	6/21 (28.6%)	8/27 (29.6%)	9/27 (33.3%)	0.634
Nakatani classification, n (%)					
Type 1	0/23 (0.0%)	0/21 (0.0%)	1/27 (3.7%)	1/27 (3.7%)	0.645
Type 2	4/23 (17.4%)	5/21 (23.8%)	5/27 (18.5%)	2/27 (7.4%)	0.467
Type 3	19/23 (82.6%)	16/21 (76.2%)	21/27 (77.8%)	24/27 (88.9%)	0.649
Posterior fixation	0/23 (0.0%)	0/21 (0.0%)	27/27 (100%)	27/27 (100%)	<0.001
Direct rami fixation	0/23 (0.0%)	21/21 (100%)	0/27 (0.0%)	27/27 (100%)	<0.001
Perioperative data—Mean ± SD					
ICU care duration, days	12.43 ± 11.98	7.62 ± 5.12	14.37 ± 16.19	11.96 ± 11.78	0.258
Ventilator care duration, days	4.52 ± 5.94	4.05 ± 6.55	5.15 ± 6.62	6.30 ± 9.95	0.948
Length of admission, days	42.87 ± 34.59	54.76 ± 45.94	41.74 ± 35.13	45.26 ± 38.18	0.495
Time to surgery, days	3.61 ± 2.48	3.30 ± 2.27	4.15 ± 4.00	4.56 ± 3.78	0.699
Radiologic reduction quality, n/N (%)					
Excellent	4/23 (17.4%)	9/21 (42.9%)	6/27 (22.2%)	7/27 (25.9%)	0.139
Good	17/23 (73.9%)	11/21 (52.4%)	20/27 (74.1%)	13/27 (48.1%)	
Fair	2/23 (8.7%)	1/21 (4.8%)	1/27 (3.7%)	6/27 (22.2%)	
Poor	0/23 (0.0%)	0/21 (0.0%)	0/27 (0.0%)	1/27 (3.7%)	
Acceptable (Good/Excellent)	21/23 (91.3%)	20/21 (95.2%)	26/27 (96.3%)	20/27 (74.1%)	0.050 *

Values are presented as mean ± standard deviation or n/N (%), as appropriate. *p*-values refer to overall comparisons among the four groups (Kruskal–Wallis test for continuous variables; chi-square or Fisher’s exact test for categorical variables). APC, anteroposterior compression; BMI, body mass index; FAST, focused assessment with sonography for trauma; ICU, intensive care unit; ISS, Injury Severity Score. Tornetta classification: Excellent, anatomic reduction; Good, <2 mm displacement; Fair, 2–5 mm displacement; Poor, >5 mm displacement. Acceptable defined as Good or Excellent. * *p*-value for acceptable rate (Fisher’s exact test, Group 3 vs. Group 4); overall 4-group chi-square *p* = 0.139. Group 1: symphyseal plating alone; Group 2: symphyseal plating + direct rami fixation; Group 3: symphyseal plating + iliosacral screw fixation; Group 4: symphyseal plating + direct rami fixation + iliosacral screw fixation.

**Table 6:** Comparison of Clinical and Radiologic Outcomes among the Four Fixation Strategy Groups.

Variable	Group 1 (*n* = 23)	Group 2 (*n* = 21)	Group 3 (*n* = 27)	Group 4 (*n* = 27)	*p*-Value
Pairwise comparisons—effect of direct rami fixation					
Operation time, min					
G1 vs. G2	64.91 ± 27.75	106.90 ± 20.83			<0.001
G3 vs. G4			95.11 ± 36.09	153.52 ± 53.71	<0.001
Nonunion, n/N (%)					
G1 vs. G2	3/23 (13.0)	3/21 (14.3)			1.000
G3 vs. G4			5/27 (18.5)	8/27 (29.6)	0.526
Implant-related complications, n/N (%)					
G1 vs. G2	5/23 (21.7)	4/21 (19.0)			1.000
G3 vs. G4			6/27 (22.2)	7/27 (25.9)	1.000
Overall complications, n/N (%)					
G1 vs. G2	7/23 (30.4)	7/21 (33.3)			1.000
G3 vs. G4			8/27 (29.6)	9/27 (33.3)	1.000
Other clinical outcomes—Mean ± SD (Kruskal–Wallis, all 4 groups)					
ICU care duration, days	12.43 ± 11.98	7.62 ± 5.12	14.37 ± 16.19	11.96 ± 11.78	0.258
Ventilator care duration, days	4.52 ± 5.94	4.05 ± 6.55	5.15 ± 6.62	6.30 ± 9.95	0.948
Length of admission, days	42.87 ± 34.59	54.76 ± 45.94	41.74 ± 35.13	45.26 ± 38.18	0.495
Time to surgery, days	3.61 ± 2.48	3.30 ± 2.27	4.15 ± 4.00	4.56 ± 3.78	0.699

Values are presented as mean ± standard deviation or n/N (%), as appropriate. Pairwise comparisons (Group 1 vs. Group 2 and Group 3 vs. Group 4) were performed using the Mann–Whitney U test for continuous variables and Fisher’s exact test for binary outcomes. Overall comparisons used the Kruskal–Wallis test. ICU, intensive care unit. Group 1: symphyseal plating alone; Group 2: symphyseal plating + direct rami fixation; Group 3: symphyseal plating + iliosacral screw fixation; Group 4: symphyseal plating + direct rami fixation + iliosacral screw fixation.

## Data Availability

The datasets used and/or analyzed during the current study are not publicly available due to institutional restrictions; however, they may be available from the corresponding author on reasonable request, subject to IRB and institutional approval.
